# Supplementary material for: Pegmatite lithium deposits formed within low-temperature country rocks
Source: Nat Commun. 2025 Jan 8;16:447. doi: 10.1038/s41467-024-55793-8 (PMC11711779; doi:10.1038/s41467-024-55793-8)
Supplement: Supplementary file 2 — Description of Additional Supplementary Files [file 41467_2024_55793_MOESM2_ESM.pdf]

Description of Additional Supplementary Files

Supplementary Data 1 - Whole-rock major and trace element compositions for rocks in the Jiajika pegmatite depoist reported in this study.
